# Erratum

**DOI:** 10.11606/s1518-8787.20200540095err

**Published:** 2020-09-24

No artigo “ **Valores de referência para plumbemia em população urbana** ”, com número de DOI: http://dx.doi.org/10.1590/S0034-89101997000200007 , publicado no periódico Revista de Saúde Pública. 1997; 31(2): 144-8, nas páginas 144, 145, 146, 147 e 148:

**Onde se lia:**

Monica M. B. Paolielo

PAOLIELO, M. M. B. et al

Monica M. Bastos Paolielo

PAOLIELO, Monica M. B.

**Leia-se:**

Monica M. B. Paoliello

PAOLIELLO, M. M. B. et al

Monica M. Bastos Paoliello

PAOLIELLO, Monica M. B.

In the article “ ***Valores de referência para plumbemia em população urbana*** ”, doi http://dx.doi.org/10.1590/S0034-89101997000200007 , published by Revista de Saúde Pública. 1997; 31(2): 144-8, in pages 144, 145, 146, 147, and 148:

**Where you read:**

Monica M. B. Paolielo

PAOLIELO, M. M. B. et al

Monica M. Bastos Paolielo

PAOLIELO, Monica M. B.

**You should read:**

Monica M. B. Paoliello

PAOLIELLO, M. M. B. et al

Monica M. Bastos Paoliello

PAOLIELLO, Monica M. B.

